# Multi-sensor-based rock glacier detection over Sikkim Himalaya

**DOI:** 10.1016/j.isci.2026.115983

**Published:** 2026-06-14

**Authors:** Ajay Kumar, Apoorva Malviya, Gulab Singh

**Affiliations:** 1 Indian Institute of Technology Bombay, Mumbai, India; 2 Indian Institute of Remote Sensing, ISRO, Dehradun, India

**Keywords:** Earth sciences, Glacial landscapes, Environmental science

## Abstract

Rock glaciers (RGs) are important landforms that store subsurface ice and influence mountain water resources; yet, their distribution in the Eastern Himalaya (EH) remains limited. This study maps RGs in the Sikkim, EH using Sentinel-1 small baseline subset (SBAS) InSAR time series (2022–2024) combined with high-resolution optical validation following the RG Inventories and Kinematics guidelines. SBAS-derived deformation signals were enhanced using seasonal-trend decomposition using LOESS (STL), which isolates long-term displacement while confining snowfall and moisture-related artifacts to seasonal and residual components. Glacier-covered areas were masked using RGI boundaries, and all candidates were validated using Google Earth Pro. A stable-moving point assessment confirmed millimeter-level stability at the reference site and ∼35 mm line-of-sight (LOS) displacement at an active RG. We identified 626 active RGs with LOS displacement rates of approximately −500 to +600 mm yr^−1^. The results refine existing RG estimates for Sikkim and demonstrate the effectiveness of SBAS InSAR with STL filtering for robust RG detection and cryosphere assessment in the EH.

## Highlights

• SBAS InSAR time series used to map rock glaciers in the Sikkim Himalaya
• STL decomposition isolates long-term deformation from seasonal signals
• ∼626 active rock glaciers identified across the Sikkim eastern Himalaya
• Stable bedrock pixels define a noise floor (σ ≈ 3.06 mm) and threshold

## Graphical abstract

**Figure:**
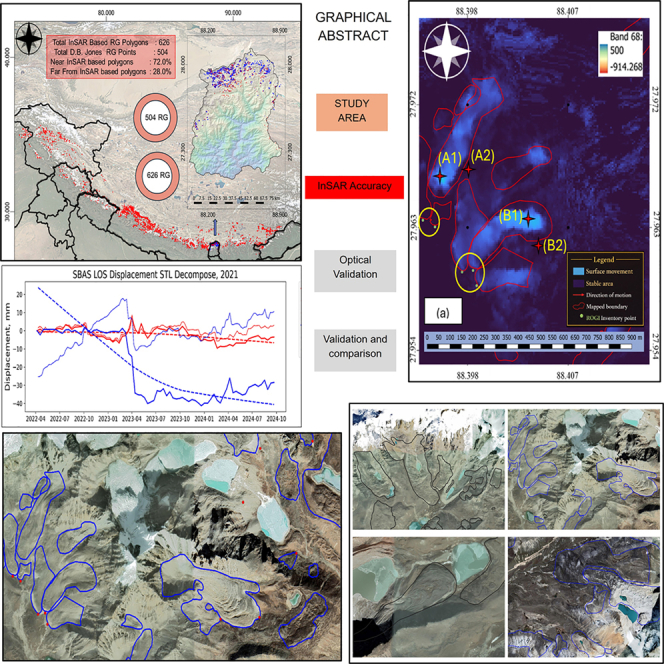

## Introduction

High-mountain Rock glaciers (RGs) mediate cryosphere, hydrosphere connection, control runoff timing, and support slope stability.^1^ RGs, which are lobate to tongue-shaped ice-debris complexes, are among its most noticeable landforms. They slide downslope due to gravity.^2^ They serve as long-lived due to “hidden” water reserves that release meltwater during dry spells and are somewhat resistant to heat due to debris insulation^3^^,^^4^. In the Himalaya, where glacier retreat is rapid, this role is particularly relevant.^5^ Research has highlighted the significance of rock-glaciers for hydrology and as markers of the occurrence of RGs in mountainous areas^1^^,^^4^^,^^6^ Kääb et al.^7^ and Bodin et al.^8^ reported active creep and its implications for slope stability in the European Alps, whereas Selley et al.^9^ emphasized the climatic vulnerability of the Andes. Sikkim has 504 RGs, according to regional inventories of the Himalaya.^3^,^4^ However, because RGs are difficult to discern between active and relict forms, prolonged cloud cover, and delicate geomorphic expression, optical-based mapping may undercount them. There are several approaches, including thermal and ground-temperature modeling, optical/geomorphic mapping,^10^ geophysical surveys,^11^ and increasingly Geodetic SAR. However, each has limitations, such as field campaigns from access limits in steep terrain, thermal models from sparse *in situ* data, and optical surveys that are hampered by cloud or snow cover uncertainties persist in regional inventories due to these limitations. For example, Raghavendra and Geetha Priya (2025)^12^ identified 223 sites, while one study^4^ reported 504 sites in Sikkim; nevertheless, many features remain unmapped or still have uncertain activity status. To address these gaps, we developed an InSAR-based detection approach that leverages Sentinel-1A’s all-weather capability and the temporal resolution of small baseline subset (SBAS) line-of-sight (LOS) time series. To isolate considerable creep, we use a conservative threshold of around ±5 mm yr^−1^ and detect deformation in the radar line-of-sight. To prevent confusion with glaciers covered in debris, RGI boundaries are used to conceal glaciated land. To guarantee geomorphic consistency (ridges-furrows, lobate/tongue morphology, and frontal steepening), candidate deformation patches are improved using high-resolution Google Earth Pro imagery. The resulting polygon inventory is then compared to Jones et al.’s independent point inventory (2021). Stable versus moving point time-series comparisons (trend only; seasonal components eliminated) are used to further verify accuracy, confirming mm-level stability at reference locations and consistent LOS deformation at active A kinematics informed inventory of 626 RGs in Sikkim is produced by this combination SBAS-InSAR-and-optical method, which enhances delineation, eliminates potential dormant forms, and permits direct comparison with historical, point-based inventories. The process offers a repeatable foundation for long-term monitoring of the eastern Himalaya’s mountain RGs and activity-based mapping.

### Study area

**Figure 1:**
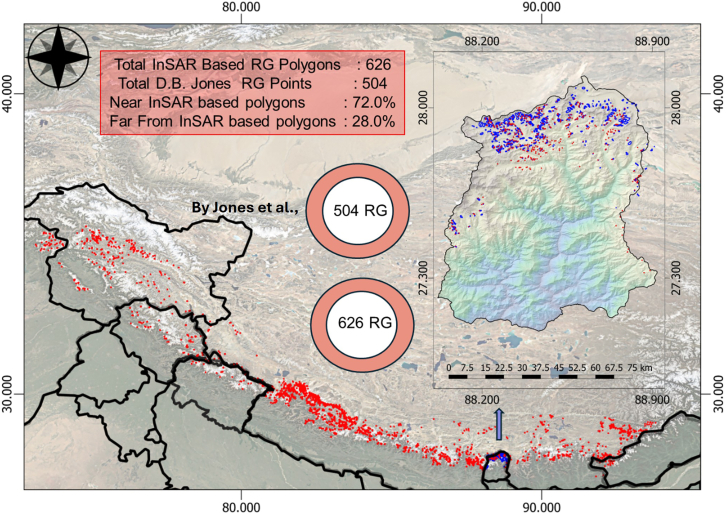
Study area and datasets Sikkim, eastern Himalaya, with state boundaries from DIVA-GIS and a hill-shade from Open Topography. The map highlights two rock-glacier inventories: InSAR-based detections (626 polygons) and (504 point) in Sikkim extracted from the red points on map generated by from Hewitt.^4^ The Himalayan-scale figure shows regional context; the Sikkim inset details spatial correspondence between datasets within Sikkim.

Sikkim Himalaya (27–28°N, 88–89°E) spans ∼7,300 km^2^ with elevations from ∼300 m to 8,586 m (Kanchenjunga), as shown in Figure 1. The region is shaped by the Indian summer monsoon which brings intense summer precipitation, while the high north-western sector experiences a cold and arid alpine climate. Permafrost is expected mainly above ∼5,000 m, strongly influenced by slope aspect and shading. The cryosphere comprises ∼84 glaciers (∼440 km^2^) and numerous glacial lakes within the Teesta basin. However, RGs in Sikkim remain largely undocumented and providing the rationale for focusing on high-elevation terrain above 4,500 m.

### Materials

**Figure 2:**
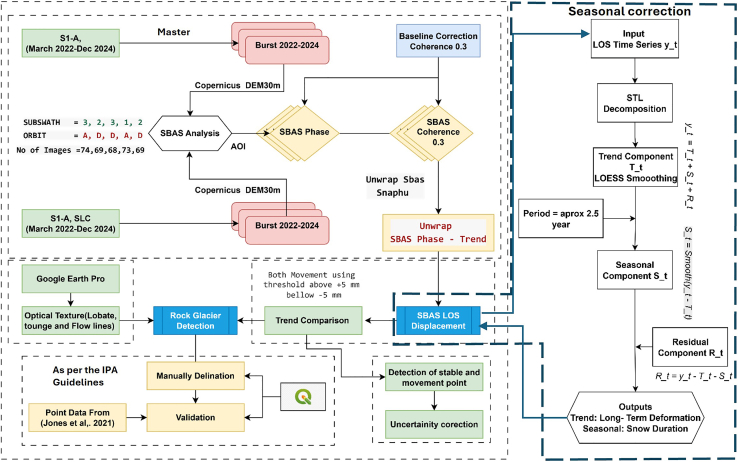
Flowchart of the SBAS-InSAR processing pipeline used to identify active RGs The diagram outlines the sequential steps beginning with Sentinel-1 SAR data preprocessing and interferogram generation, followed by SBAS time-series inversion and STL (Seasonal-Trend decomposition using Loess) filtering to separate long-term deformation from seasonal snow/rainfall effects and atmospheric noise. The final outputs include trend-based displacement maps and time-series curves used for validation and identification of active rock-glacier motion.

Several datasets were used to detect and validate RG activity for the methadology (Figure 2) in the study. Table 1, summarizes the data sources, types, temporal coverage, spatial resolution, and the purpose of each dataset.

**Table 1:** Dataset used in the study

Dataset	Data source and reference	Type	Temporal coverage	Spatial resolution
Sentinel-1A SAR (Asc. and Desc.) to cover entire Sikkim	European Space Agency (Copernicus Sentinel-1)	C-band SAR imagery (IW SLC)	Apr 2022–Sep 2024 (∼12-day revisit)	5 × 20 m
Copernicus DEM (30 m)	European Space Agency (Copernicus Program)	Digital Elevation Model (DEM)	2020	30 m
Google Earth Pro Imagery	Google/Digital	High-resolution optical imagery	2018–2023 (various)	∼1 m (varies)
Rock Glacier Inventory^4^	Jones et al.^4^	Point locations of RG’s	Compiled 2021 (static)	N/A (point data)

The Sentinel-1A SAR data consist of interferometric wide swath, single look complex images acquired in both ascending and descending orbits over the study area as shown in Table 2. A total of approximately 70 acquisitions per orbit direction were obtained between April 2022 and September 2024, providing a ∼2.5-year time series of observations. The Copernicus 30 m DEM (GLO-30) was used^13^ to assist in InSAR processing by removing the phase contribution due to topography (differential InSAR) and to geocode results.^14^^,^^15^^,^^16^^,^^17^ High-resolution satellite imagery available in Google Earth Pro (sub-meter to 1 m resolution) provided context for identifying geomorphological features of RGs and was used for validation and mapping refinement. Additionally, an existing inventory of RGs from^4^ comprising point coordinates of mapped RG fronts in the region was employed as ground truth for validation of the InSAR-detected active RGs.

**Table 2:** Number of Bursts and duration

Dataset	Orbit	Swath	No of images	Duration
Sentinel 1A	A	IW-3 burst	74	03/04/2022–07/09/2024
Sentinel 1A	D	IW-2 burst	69	06/04/2022–10/09/2024
Sentinel 1A	D	IW-3 burst	68	06/04/2022–10/09/2024
Sentinel 1A	A	IW-1 burst	73	03/04/2022–14/09/2024
Sentinel 1A	D	IW-2 burst	69	06/04/2022–07/09/2024

## Results

### Rock glacier boundary delineation

**Figure 3:**
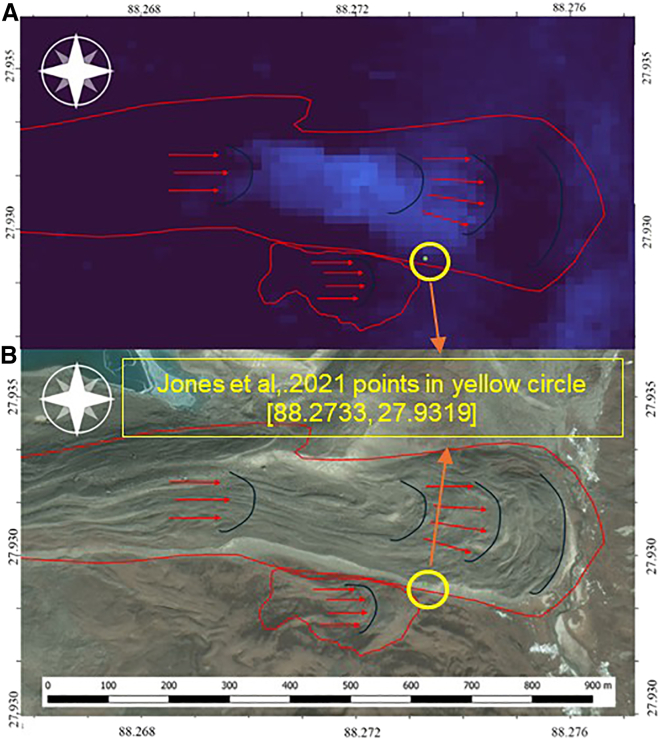
Comparison of RG morphology and surface movement patterns Derived from two approaches: (A) InSAR-based displacement map showing zones of deformation within the glacier body and (B) optical imagery from Google Earth Pro.

To delineate the RGs from the InSAR data, we created the polygons by grouping contiguous zones of significant LOS motion. Each polygon initially corresponded to a cluster of pixels exceeding the ±5 mm/yr threshold, essentially outlining the moving “core” of a potential RG. Then, we refined these preliminary polygons using high-resolution optical imagery form Google Earth Pro. In this step, the boundaries of each RG were adjusted to align with the visible geomorphological extent of the RG landform. For example, Figure 3 shows an instance of an InSAR-derived hotspot overlain on Google Earth imagery. The zone of LOS-detected movement coincides with a tongue-shaped, debris-covered feature exhibiting a steep front and surface lineation. We expanded or trimmed the polygon in such cases to match the full lobate outline of the RG as seen from above. This process ensured that the mapped polygons encompass the entire RG (from the root zone to the frontal margin) rather than just the portion displaying motion.

**Figure 4:**
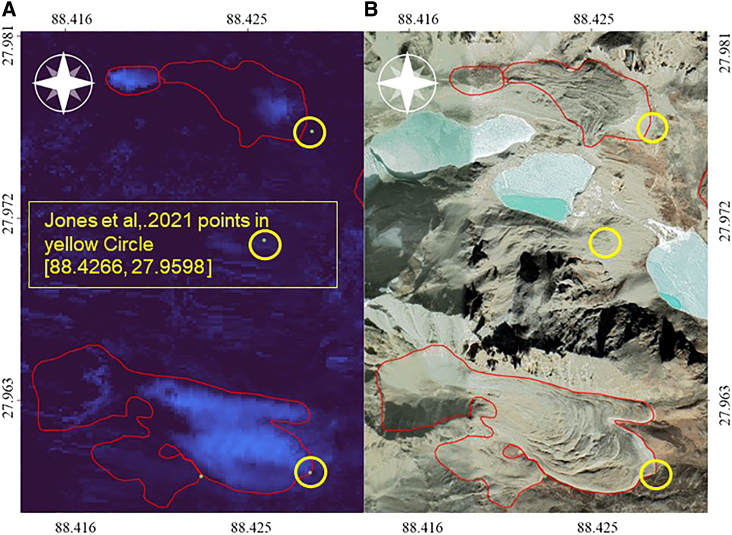
Comparison of RG morphology and surface movement patterns Derived from two approaches: (A) InSAR-based displacement map showing zones of deformation within the glacier body and (B) high-resolution optical imagery from Google Earth.

It also helped in excluding any pseudo-InSAR signals (e.g., from adjacent slopes or radar shadow) by confirming the presence of a real landform. After refinement, the final inventory consists of 626 polygons, representing individual RG of varying size. The refinement with time series Google Earth imageries also allowed us to categorize the morphology where possible. For instance, some polygons were noted as glacier-derived RGs (originating in former glacial cirques) versus talus-derived ones, based on their position and shape, although a full genetic classification is beyond this results scope. Overall, the RG boundary delineation step (Figure 4) provided a crucial bridge between the InSAR data and a tangible RG inventory resulting in well-defined outlines for subsequent analysis.

### Validation with optical imagery

**Figure 5:**
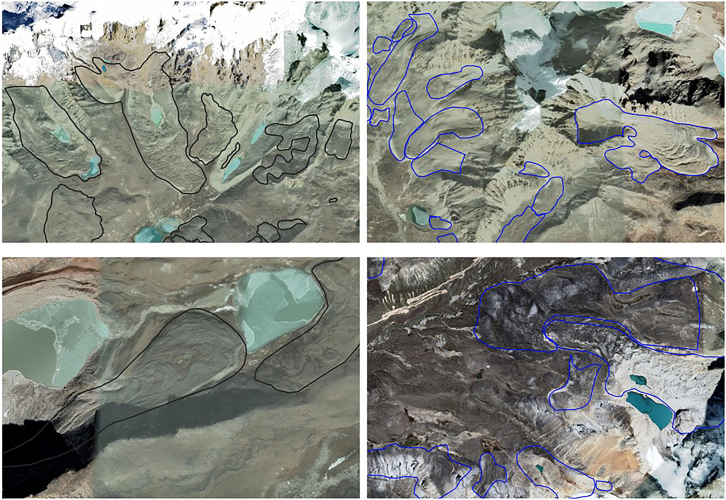
Examples of mapped RGs in the study area using high-resolution optical satellite imagery The outlines (black and blue polygons) delineate RG bodies of varying size and morphology, highlighting their spatial distribution across different valleys and mountain slopes.

To ensure that the InSAR-derived deformation fields correspond to actual RGs, we conducted a morphological validation using high-resolution optical imagery in QGIS. Each polygon delineating a moving area was overlain on Google Satellite imagery and inspected for classic RG landform characteristics. These characteristics include well-developed surface ridges and furrows, a steep pronounced front (frontal steepening), and a tongue-like or lobate planform shape. RGs typically exhibit a distinct flow texture, for example, sets of transverse ridges and troughs resulting from creeping ice-debris, and “swollen” lobate bodies with steep margins that differentiates them from other landforms as shown in Figure 5. Using these criteria, we verified that each InSAR polygon indeed covers a geomorphologically plausible RG. In QGIS we validated, most of the polygons corresponded to debris-covered slopes with the expected tongue-shaped outline and ridge furrow pattern visible on the satellite baseman, reinforcing that our InSAR method is detecting true RGs. For any ambiguous InSAR signals, we performed cross-comparison with the optical baseman to rule out false positives. In some instances, deformation signals could originate from non-RG processes such as slow-moving landslides, glacier terminus dynamics, or other mass movements. When an InSAR hotspot lacked the clear surface morphology of a RG, we treated it with caution. For example, if a moving area was located on a smooth vegetated hillside or adjacent to an active glacier toe, the high-resolution google earth imagery helped identify it as a likely landslide or glacier-related motion rather than a RG. Such features were excluded from the final inventory. This integrated approach follows recommendations in the literature that emphasize combining InSAR with optical interpretation to improve RG inventories.

### Comparison and validation with existing inventory

We compared the InSAR-based RG outlines with the independent point inventory of Jones et al.^4^ for Sikkim. Our SBAS InSAR mapping produces polygons representing actively deforming RG surfaces, whereas the validation dataset encodes each landform as a single point (typically near the toe). Only a limited number of validation points coincide strictly inside the InSAR polygons; most lie just outside the mapped outlines. This mismatch reflects (i) differences in delineation methodology (polygon vs. point representation) and (ii) the addition of new, actively deforming features not captured by previous, optics-only inventories. Similar discrepancies between polygon-based mapping and point inventories have been reported elsewhere, given variable mapping criteria and the inherent difficulty of identifying RGs from optical imagery alone.^4^^,^^8^^,^^10^

**Figure 6:**
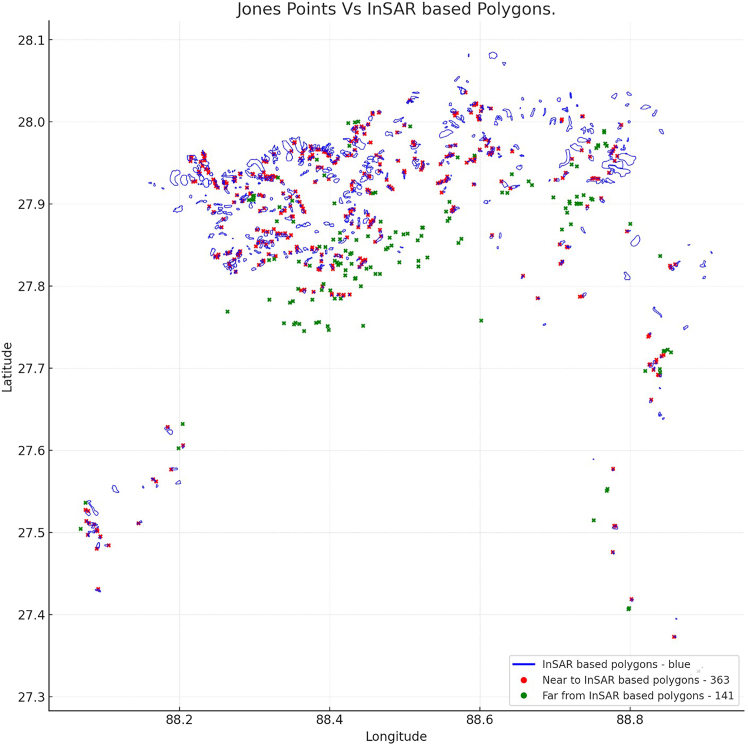
Overview map. InSAR-based RG polygons are shown as blue outlines*-*^4^ Points within 300 m of a polygon are red (“near to InSAR based polygons – 363”), and those farther than 300 m are green (“far from InSAR based polygons – 141”). Title: Jones points Vs. InSAR-based polygons. This map emphasizes that many legacy points clusters near (but not always within) actively deforming polygons, consistent with small geolocation offsets, differences in mapping conventions, or points placed at the distal tip.

**Figure 7:**
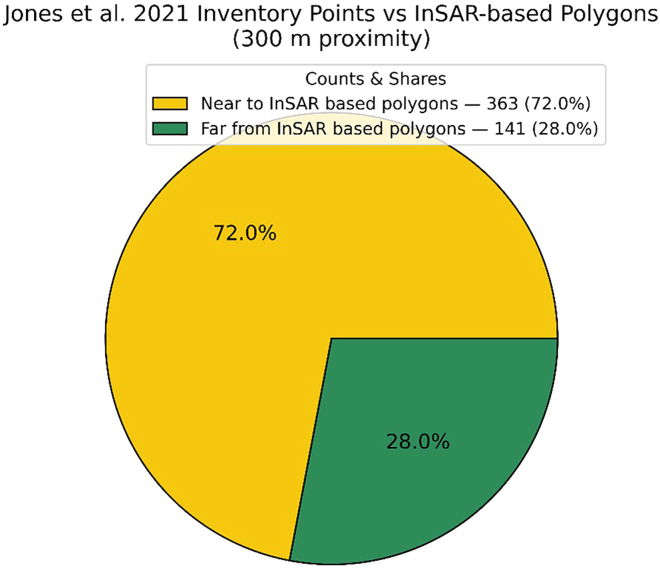
Near far pie (300 m) Using the same 300 m criterion, ∼72.0% of Jones points (363/504) are “near” our polygons, while ∼28.0% (141/504) are “far,” indicating broad spatial association even when strict inclusion is not met.

We also quantified strict and tight-buffer overlap for completeness at a 300 m proximity threshold (Figures 6 and 7), 72.0% of Jones points fall near our InSAR polygons (363, red) and 28.0% are far (141, green), consistent with polygon-point representation differences and modest spatial offsets. These values show partial correspondence but also underscore that many legacy points likely mark intact but inactive/relict landforms that exhibit no current creep—a known limitation of morphology which only inventories.^4^^,^^8^^,^^10^ Inventory outcome and validation done by applying a conservative ±5 mm yr^−1^ LOS threshold to SBAS time series and refining with Google Earth Pro geomorphic checks yields 626 InSAR-detected rock-glacier polygons in Sikkim. Validation against the^4^ points (as summarized above) and stable vs. moving point time-series comparisons (seasonal component removed) confirm that our detections represent actively deforming ice-debris complexes, while stable references remain near zero trend supporting both the detection specificity and the LOS velocity fidelity. Decorrelation in monsoon/vegetated terrain, dense vegetation, wet snow, and seasonal moisture swings can reduce coherence, obscuring motion signals. We mitigated this with SBAS temporal averaging, coherence weighting, and a conservative activity threshold. Geometry effects (layover/shadow and LOS projection), steep relief can cause radar layover/shadow, and deformation not aligned with the radar line-of-sight may be underestimated. Using both ascending/descending geometries (where available) and focusing on spatially consistent creep patches helps reduce false negatives. Atmospheric/orbital artifacts, stratified troposphere and long-wavelength ramps can bias trends. We used regression-based trend correction (polynomial in x–y–H) to remove height-correlated atmosphere and orbital ramps before classification. Short record lengths, a 2022–2024 window may miss interannual variability. We adopt a conservative ±5 mm yr^−1^ threshold and corroborate with independent optical checks to limit false positives. InSAR LOS time-series + optical validation provides a kinematics-informed inventory that distinguishes active from inactive RGs. The new 626 polygon inventory extends and refines prior optics which only counts for Sikkim, while our near or far and overlap metrics which explains expected discrepancies between polygon mapping and point inventories.

Geometric distortion, observational completeness, and inventory bias (layover/shadow and LOS projection), steep relief in Sikkim can cause radar layover and shadow that render portions of terrain locally invisible to InSAR regardless of processing choices, and deformation not aligned with the radar line-of-sight may be underestimated or remain undetected.^18^^,^^19^ In mountainous terrain, layover occurs when the radar signal from a higher elevation point arrives at the sensor before that from a lower point, compressing or inverting the signal, while shadow arises on slopes facing away from the satellite where no radar return is received at all.^20^^,^^21^ Both effects are geometrically deterministic functions of terrain slope, aspect, and satellite incidence angle, and represent inherent limitations of any InSAR analysis in high-relief regions such as the Eastern Himalaya.^22^^,^^23^

To minimize this limitation, the present study uses both ascending and descending Sentinel-1 acquisition geometries, which observe the terrain from opposite look directions; areas affected by radar shadow or layover in one geometry are frequently visible in the other, substantially improving terrain coverage and reducing systematic observational bias relative to single-geometry studies.^21^^,^^24^^,^^25^ This dual-geometry strategy is now considered standard practice in InSAR-based RG inventories over steep alpine terrain.^24^^,^^26^ Additionally, focusing on spatially consistent creep signatures across multiple interferograms rather than isolated phase patterns in single interferograms further reduces geometry-driven false negatives.^21^^,^^27^

**Figure 8:**
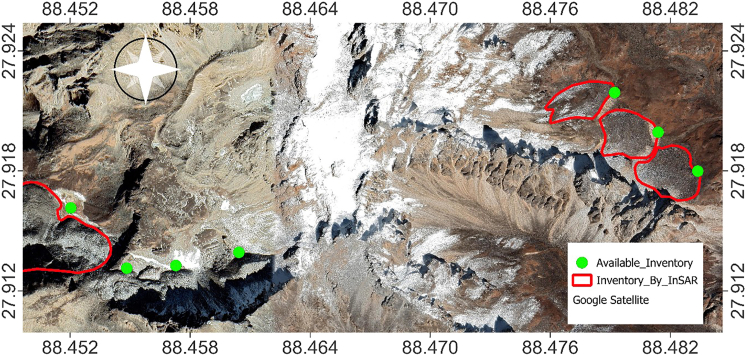
Spatial correspondence between SBAS-InSAR-derived RG polygons (red outlines; Inventory_By_InSAR) and independent optical reference inventory points (green dots; Available_Inventory) in northern Sikkim (∼27.917°N, 88.467°E) Red polygons delineate actively deforming units identified by SBAS-InSAR; green dots represent reference locations from Harrison et al. and Jones et al.,^4^ independent of SAR viewing geometry. Reference points outside InSAR polygon boundaries correspond to either relict or inactive landforms below the ±5 mm yr^−1^ detection threshold, or features suppressed by radar geometric distortion or vegetation-driven decorrelation. Background, Google Satellite imagery, Google.

To empirically quantify the impact of these geometric limitations on inventory completeness, we compared our InSAR detections with the independent reference inventory of Jones et al.,^4^ both compiled from optical remote sensing and geomorphic interpretation, and therefore, entirely unaffected by SAR viewing geometry constraints. Such cross-comparison between kinematic InSAR inventories and independently derived optical inventories represents the most direct empirical test of detection completeness available in the absence of exhaustive field surveys;^24^^,^^28^ this spatial correspondence is illustrated in Figure 8, which shows a representative sub-region where InSAR-derived polygons (red outlines) overlap with independent optical inventory points (green dots); the majority of reference points fall within or immediately adjacent to detected InSAR polygons, confirming successful observational coverage of actively deforming terrain across the study domain.

Of the total reference inventory features within the study region, approximately 15–16 RGs were not detected in the present InSAR-based analysis. These undetected features can be attributed to two distinct and separable mechanisms. First, approximately half are situated in steep, deeply incised valley walls and north-to northwest-facing terrain where radar geometric distortion specifically shadow and layover locally suppresses interferometric coherence in both the ascending and descending geometries, rendering these features permanently invisible to InSAR irrespective of the processing approach adopted;^20^^,^^24^ north-facing slopes are well established as the preferential aspect class for active RGs across the Himalaya, as shading suppresses ablation of the internal ice matrix and sustains permafrost conditions necessary for long-term creep;^29^^,^^30^^,^^31^ the coincidence of radar shadow with this thermally favored aspect class, therefore, represents a systematic but spatially bounded observational bias that affects all satellite SAR-based inventories in the region. Second, the remaining undetected features occur at lower elevations below approximately 4,500 m a.s.l. where dense vegetation cover, wet soil conditions, and high seasonal moisture variability during and following the Indian summer monsoon cause significant temporal decorrelation of the SAR signal, substantially reducing interferometric coherence and masking any underlying surface deformation signal.^32^^,^^33^^,^^34^ Reference points falling outside InSAR polygon boundaries in these lower-elevation zones are, therefore, interpreted as features suppressed by vegetation-driven decorrelation rather than as inactive or relict landforms, and their absence from the inventory reflects an elevation-dependent observational constraint rather than a kinematic classification.^4^^,^^8^^,^^10^

We, therefore, explicitly acknowledge that our 626-polygon inventory constitutes a minimum estimate subject to two distinct spatial constraints: (i) a geometry-driven gap concentrated on steep north-facing slopes and deeply incised valleys where radar shadow and layover are irreducible within the Sentinel-1 imaging configuration, and (ii) a vegetation-driven gap at lower elevations where monsoon-season decorrelation suppresses coherence. The inventory is considered spatially complete for all terrain that is both observable in at least one SAR geometry and sufficiently coherent for SBAS time-series analysis.

### Removal of snow and precipitation artifacts from InSAR time series

**Figure 9:**
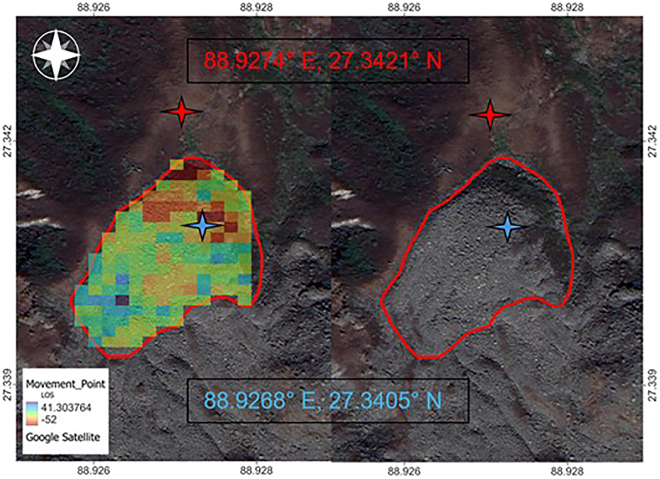
SE-Sikkim location of reference points used for SBAS-InSAR validation The red symbol denotes the stable reference site (88.9274, 27.3421), while the blue symbol marks the moving RG site (88.9268, 27.3405).

**Figure 10:**
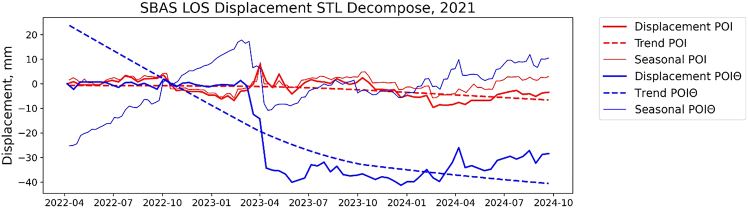
SBAS-InSAR LOS displacement time series for the stable reference point (red curve) and the moving RG point (blue curve) from April 2022 to October 2024 STL-derived long-term trends are shown in figure, with seasonal components removed for clarity. These results demonstrate that STL filtering substantially enhances the reliability of SBAS-InSAR deformation estimates in snow-dominated Himalayan terrain.

**Figure 11:**
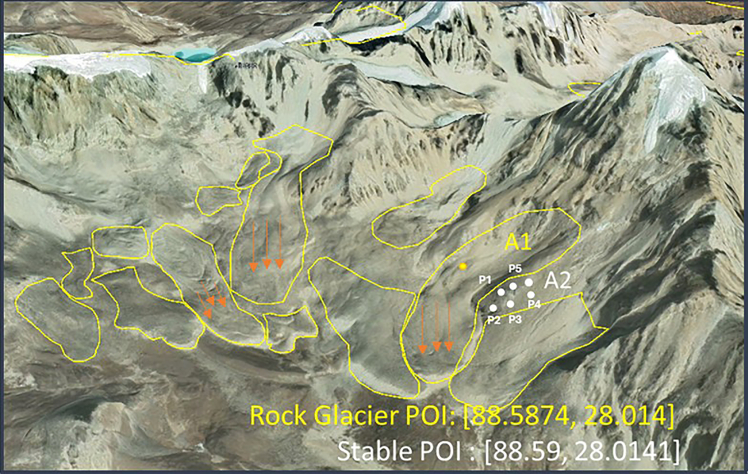
North Sikkim three-dimensional Google Earth Pro view of the study area showing mapped RG polygons (yellow outlines), inferred downslope movement directions (orange arrows), and the locations of the moving (A1) and stable (A2) reference points with five stable points on hard rock P1, P2, P3, P4, and P5 Inferred downslope movement directions (orange arrows), and the locations of the moving (A1) and stable (A2) reference points with five stable points on hard rock P1, P2, P3, P4, and P5.

The SBAS-InSAR LOS displacement time series initially show short-term fluctuations coinciding with periods of intense snowfall and precipitation, which particularly occurs during the winter months (December–January). These fluctuations are characteristic of snow-induced decorrelation, wet-snow loading, surface moisture changes, and transient atmospheric phase delays, which commonly affect InSAR observations in high-altitude Himalayan environments. Application of STL (seasonal-trend decomposition using LOESS) As shown in Figures 9 and 10 effectively separates these snow and precipitation-related disturbances from the deformation signal over south-east (SE) RG. As shown in Figure 11, the seasonal and residual components capture short-duration variability associated with snowfall and precipitation, while the long-term trend component remains smooth and temporally coherent. Winter-season anomalies visible in the raw displacement time series are absent from the STL-derived trend, indicating successful mitigation of snow-related artifacts. The filtered trend, therefore, represents deformation that is independent of seasonal snow cover and precipitation effects. This improvement is particularly evident during the winter periods, where raw time series exhibit increased scatter, but the STL trend remains continuous and stable (Figure 10).

### Validation of SBAS-InSAR deformation using stable and moving reference points

Validation of the SBAS-InSAR results was performed using a stable versus moving point comparison based on STL-filtered LOS displacement trends for the period April 2022–October 2024. The spatial locations of the reference points are shown in Figure 9, with the stable point located on non-moving terrain and the moving point situated within an actively deforming RG body (Figure 11) of north Sikkim RG. The stable reference point exhibits an essentially flat LOS displacement trend throughout the observation period, with residual variations confined to approximately ±5 mm (as in Figures 9 and 11).

**Figure 12:**
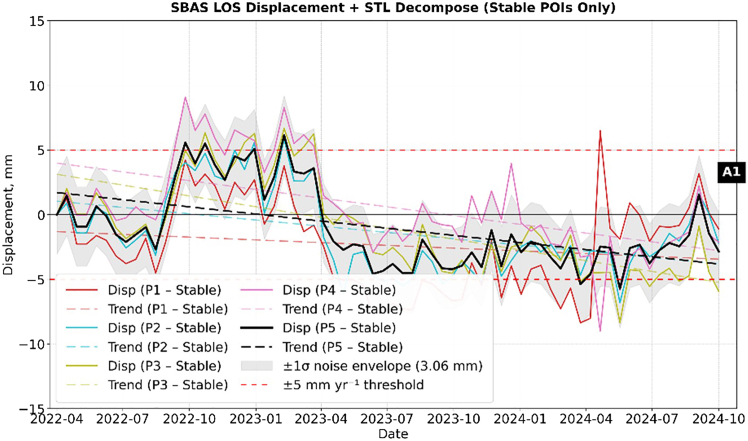
SBAS-InSAR LOS displacement time series LOS displacement time series and STL decomposition for stable reference pixels (P1–P4) and pooled pixel (P5) from January 2022 to October 2024. Gray shading indicates the ±1σ noise envelope (σ_noise = 3.06 mm); red dashed lines mark the ±5 mm yr^−1^ detection threshold (1.6σ). All stable pixels remain within the noise envelope while confirming their suitability as bedrock reference points.

**Figure 13:**
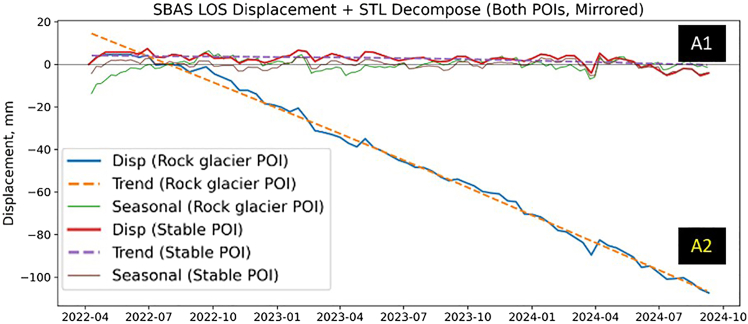
Combined SBAS LOS displacement and STL decomposition results for both reference points The RG point exhibits a strong monotonic displacement trend, while the stable point remains near zero displacement, demonstrating effective removal of snow- and precipitation-related artifacts and validating the InSAR measurements.

To provide a systematic and reproducible noise characterization in response to this concern, four stable reference pixels (P1–P4) were selected on exposed bedrock surfaces based on objective, pre-defined criteria specifically, SBAS coherence γ ≥ 0.35 maintained throughout the full observation period and no detectable surface change in multi-temporal optical imagery ensuring that the reference sample reflects stable bedrock conditions rather than a subjective manual selection. The standard deviation of LOS displacements was computed independently for each pixel and then pooled as a band-wise spatial mean (P5, *n* = 68 acquisition epochs), yielding a formal noise floor of σ_noise = 3.06 mm; the per-pixel values range narrowly from 3.14 mm (P2) to 3.62 mm (P3) as shown in Figures 12 and 13, confirming spatially consistent noise levels across the reference area (Table 3; Figure 11). The applied detection threshold of ±5 mm yr^−1^ corresponds to approximately 1.6σ above this noise floor which conservatively exceeding the background noise envelope while the rock-glacier point of interest exhibits a cumulative LOS displacement of ∼400 mm over 2.5 years (∼160 mm yr^−1^), yielding a signal-to-noise ratio of approximately 52, which unambiguously confirms that the observed displacement signal cannot be attributed to atmospheric artifacts, orbital ramps, or any other noise source characterized by the stable bedrock reference pixels.

**Table 3:** Statistical summary of SBAS-InSAR LOS displacement for stable reference pixels (P1–P4) and pooled pixel (P5; band-wise mean, *n* = 68 epochs, January 2022–October 2024)

Point	Mean (mm)	σ (mm)	RMSE (mm)	Rate (mm yr^−1^)	|d|max (mm)
P1	−2.37	3.48	4.21	−0.45	8.36
P2	−1.38	3.14	3.43	−0.88	6.81
P3	−1.05	3.62	3.77	−2.37	8.34
P4	+0.60	3.51	3.56	−0.85	9.10
P5	−1.05	3.06	3.23	−1.14	6.15
σ_noise (P5) 2σ 3σ SNR (vs. RG) Threshold		3.06 mm 6.12 mm 9.18 mm ≈52 ±5 mm yr^−1^ (∼1.6σ)			

Notation: mean = temporal mean LOS displacement; σ = standard deviation of displacement time series; RMSE = root-mean-square error; rate = mean annual LOS velocity; |d|_max_ = maximum absolute single-epoch displacement; all units in mm except rate (mm yr^−1^). The pooled noise floor σ_noise = 3.06 mm; the ±5 mm yr^−1^ detection threshold corresponds to ∼1.6σ, yielding SNR ≈52 relative to the rock-glacier POI.

**Figure 14:**
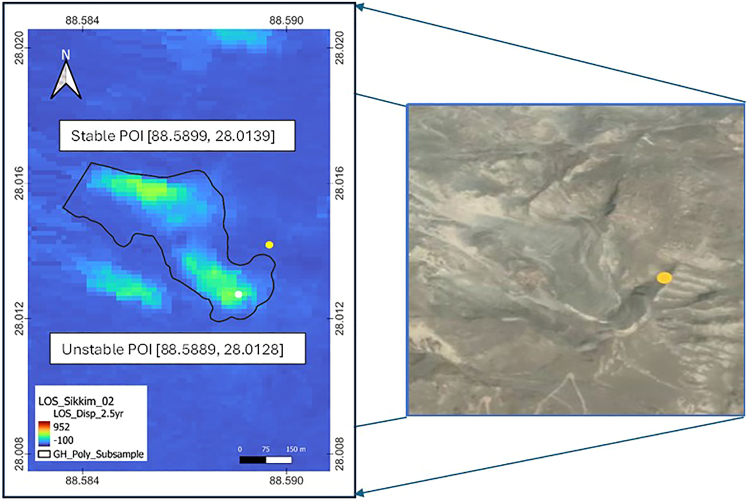
Presents an example of a RG mapped in NE Sikkim, overlaid on SBAS-derived LOS displacement patterns Overlaid on SBAS-derived LOS displacement patterns.

**Figure 15:**
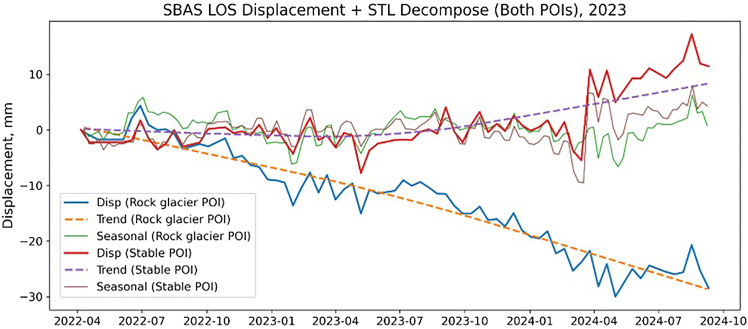
STL-filtered LOS displacement time series for representative stable (red curves) and moving RG points (blue curves) in NE Sikkim for the period April 2022–October 2024

**Figure 16:**
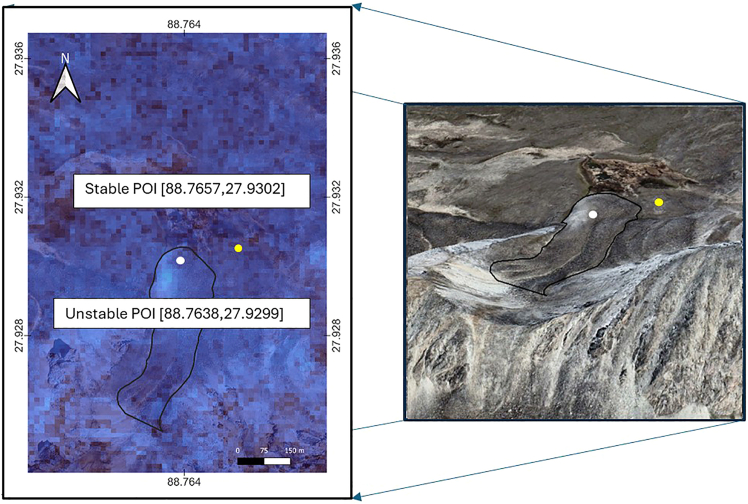
Illustrates the spatial context of the selected stable and unstable points of interest used for SBAS-InSAR validation in the north-eastern Sikkim sector The left panel shows the RG outline overlaid on a satellite basemap, with the unstable point of interest (POI) located within the central part of the RG body and the stable POI positioned on adjacent non-moving terrain. The unstable point lies within the debris-mantled lobe characterized by smooth, elongated morphology typical of active RGs, whereas the stable point is situated outside the mapped RG boundary on comparatively compact and geomorphologically inactive ground.

**Figure 17:**
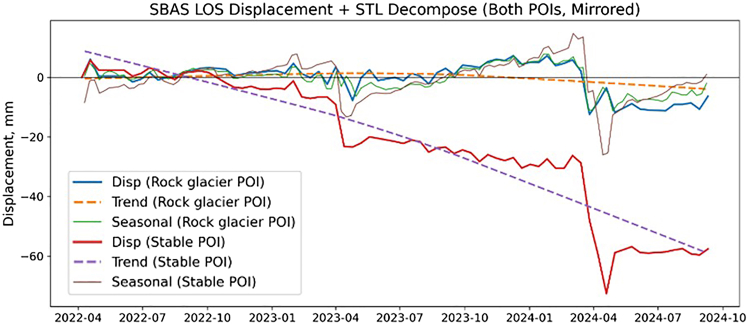
Combined SBAS LOS displacement and STL decomposition for a representative NE Sikkim RG site The moving point exhibits a strong, continuous deformation trend, while the stable point remains effectively stationary, confirming successful removal of snow- and precipitation-related artifacts and validating the InSAR measurements.

**Figure 18:**
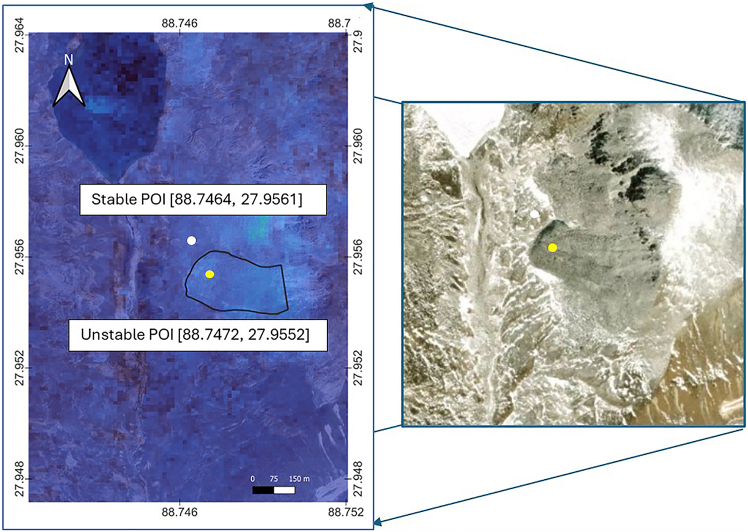
Illustrates the spatial context of the selected stable and unstable POIs used for SBAS-InSAR validation in the north-eastern Sikkim sector The left panel shows the RG outline overlaid on a satellite basemap, with the unstable POI located within the central part of the RG body and the stable POI positioned on adjacent non-moving terrain. The unstable point lies within the debris-mantled lobe characterized by smooth, elongated morphology typical of active RGs, whereas the stable point is situated outside the mapped RG boundary on comparatively compact and geomorphologically inactive ground.

**Figure 19:**
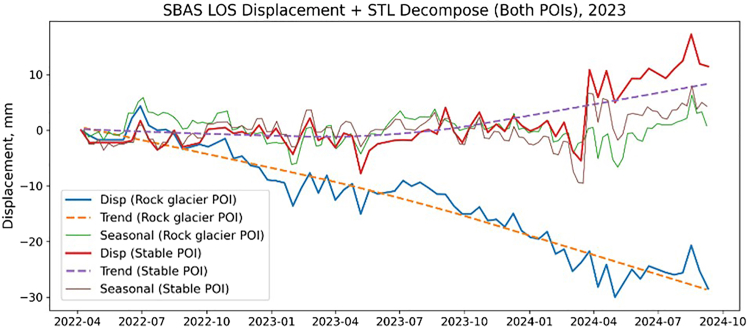
STL-filtered LOS displacement time series for representative stable (red curves) and moving RG points (blue curves) in NE Sikkim for the period April 2022–October 2024

To further evaluate the robustness of the SBAS-InSAR deformation estimates, additional validation was conducted in the north-eastern (NE) sector of Sikkim, a region characterized by higher elevations, persistent seasonal snow cover, and strong monsoonal as well as winter precipitation influence. These conditions represent one of the most challenging environments for InSAR-based deformation analysis in the eastern Himalaya. Independent validation of SBAS-InSAR results in the NE Sikkim sector. This spatial contrast indicates localized ground motion associated with the RG rather than broad-scale atmospheric or snow-related artifacts. The corresponding STL-decomposed LOS displacement time series for representative stable and moving points within this NE Sikkim site are shown in Figure 14. Prior to STL filtering, the time series exhibit short-term fluctuations related to seasonal snow accumulation and melt. Following STL decomposition Figure 15, these high-frequency variations are captured within the seasonal and residual components, while the trend component reveals a clear, monotonic deformation signal at the RG point similarly in Figures 16, 17, 18 and 19.

**Figure 20:**
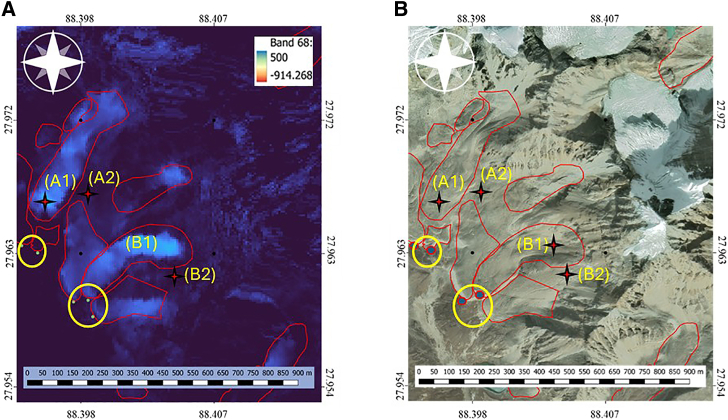
Stable Vs Unstable point comparision A1 is a Unstable point A2 is a stable point Similarly B1 is Unstable and B2 is stable point (A) Rock-glacier area of interest mapped from Google satellite imagery also shown in Figure 9. The red polygon outlines surface geomorphology (lobes, ridges, and fronts). Grid ticks are geographic (°) with north arrow and scale bars shown. (B) SBAS InSAR LOS displacement map, yellow circles representing Jones et al. points. Refer to Figure 11 for locations.

**Figure 21:**
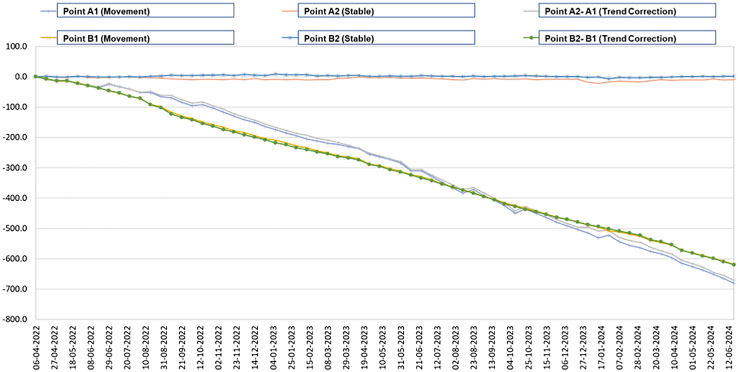
LOS time-series at A1, A2, B1, B2, and a stable point (control). The stable point exhibits only a weak linear drift and is used for uncertainty correction

The spatial distribution of deformation shows a coherent signal confined to the RG body, while the surrounding terrain exhibits relatively homogeneous and low-magnitude displacement. The RG point displays a sustained negative LOS displacement trend from April 2022 through October 2024, with cumulative deformation exceeding several tens of millimeters (Figure 15). In contrast, the stable reference point located outside the RG polygon shows a near-zero long-term trend, with residual variability confined within a few millimeters. Importantly, the persistence of the deformation trend across multiple winter seasons demonstrates that the observed signal is not driven by transient snow cover, rainfall, or freeze-thaw cycles, but reflects genuine ground motion as shown in Figures 20 and 21 and Table 4.

**Table 4:** Site-wise SBAS-InSAR validation and relative activity ranking (2022–2024)

Site	Relative activity	Visual trend	Cumulative LOS class∗	Notes
A1	Highest	Strong, steady negative slope	High	Central lobe; matches red patch on SBAS map
B1	High	Strong, steady negative slope	High	Eastern lobe; compact hotspot
A2	Moderate	Moderate negative slope	Medium	Interior area, coherent and persistent
B2	Lower	Gentle negative slope	Low-medium	Southern tongue; slower sector
Stable	Ref.	Near-flat with weak drift	+5, −5	Used for uncertainty/common-mode correction

A1 shows the highest activity with a strong, steady negative LOS slope and high cumulative class, coincident with the central red creep patch on the SBAS map; B1 is high (eastern lobe, compact hotspot); A2 is moderate (coherent, persistent signal; medium class); B2 is lower (gentle slope; low-medium class). The Stable site is near-flat within ±5 mm yr^−1^ and was used for uncertainty/common-mode correction. (Negative LOS denotes motion toward the sensor under our convention; time series shown after seasonal removal and regression-based trend correction.)

The consistency of stable-versus-moving point behavior across multiple NE Sikkim sites confirms the regional reliability and transferability of the SBAS-InSAR + STL framework. Even under conditions of persistent snow cover and high precipitation, the method successfully distinguishes active RG-controlled deformation from non-moving terrain. This independent validation from NE Sikkim provides strong support that the mapped RG deformation represents true RG creep, rather than artifacts arising from seasonal snow or atmospheric variability. The absence of systematic drift at this location confirms that residual snow, precipitation, and atmosphere related artifacts have been effectively suppressed and that the SBAS-InSAR stack is temporally stable. This stable point, therefore, provides a robust baseline for validating deformation signals across the study area. In contrast, the RG point displays a clear, monotonic LOS displacement trend after STL filtering. As illustrated in Figures 10 and 12, cumulative displacement reaches approximately ∼35 mm over the study period, corresponding to an average LOS velocity of ∼10–15 mm yr^−1^. The persistence of this trend after removal of seasonal components demonstrates that the observed deformation is not driven by snowfall, rainfall, or freeze-thaw effects, but reflects sustained ground motion. The pronounced divergence between the stable and moving point trends (Figures 9, 10, 11, and 12) provides strong validation of the SBAS-InSAR results. While both points are subject to similar climatic and atmospheric forcing, only the RG point exhibits significant long-term displacement. The stable point remains effectively stationary, confirming that the detected deformation signal is spatially localized and physically meaningful rather than an artifact of seasonal snow cover or precipitation variability. Together, these results confirm that SBAS-InSAR combined with STL decomposition can reliably resolve millimeter-scale RG deformation in high-relief, snow-affected Himalayan environments, providing high confidence in the detected RG-driven ground motion. The spatial deformation map reveals significant heterogeneity in the RG zone with movement ranging from −914 mm (away from the sensor) to +500 mm (toward the sensor). Time series analysis at two points A1 and B1 (as shown in Figure 11) demonstrates consistent subsidence rates of approximately −260 mm/year at active zones while one point remains stable, indicating localized RGs thaw and movement (Liu et al. 2020)^35^. Furthermore, other recent Himalayan InSAR applications demonstrate that rock-glacier velocities can vary by more than an order of magnitude across high-mountain Asia, which helps to frame a relatively modest Sikkim LOS rate. Hu et al.^36^ used InSAR-derived velocities as constraints for rheological modeling of five RGs in the Khumbu and Lhotse valleys (NE Nepal) and report mean downslope velocities of 5–30 cm yr^−1^ (i.e., 50–300 mm yr^−1^) over a much longer observational window (2006–2020) for the coherently moving parts. These Nepal values are substantially higher than our ∼10–15 mm yr^−1^ LOS rates in NE Sikkim, underscoring that Himalayan rock-glacier kinematics span from slow, near-threshold motion (mm-cm yr^−1^) to faster cm-dm yr^−1^ regimes, depending on site conditions and measurement geometry. Hu et al.^36^ also explicitly highlights a key comparability constraint that is equally relevant for our study which is converting 1-D LOS measurements to downslope motion requires assumptions (e.g., purely downslope creep without a vertical/subsidence component) and may be affected by LOS sensitivity limitations in complex terrain.

Therefore, when we compare our Sikkim LOS values to Nepal or high-mountain Asia downslope velocities, the most rigorous statement we can make is that Sikkim sites occupy the lower-velocity, transitional end of the Himalayan distribution, while still exhibiting the essential diagnostic hallmark used in InSAR inventories—persistent, spatially coherent deformation collocated with rock-glacier morphology.

### Integrated morphology-kinematics-topography validation framework

We adopt a systematic and reproducible validation framework that integrates geomorphological evidence from high-resolution optical imagery with kinematic evidence derived from SBAS-InSAR time-series deformation. Such combined approaches are increasingly recommended in modern rock-glacier inventories to reduce subjectivity and improve reproducibility.^24^^,^^25^^,^^28^ Rather than relying solely on visual interpretation, each InSAR-derived polygon is evaluated against explicit and sequential acceptance criteria. Firstly, a polygon is considered a rock-glacier candidate only if it exhibits diagnostic geomorphological characteristics, including a lobate or tongue-shaped debris body, downslope elongation, well-defined lateral margins, and surface textures indicative of debris-mantled ice creep. These criteria follow established geomorphological mapping guidelines;^34^^,^^37^ and recent large-scale inventories that combine optical interpretation with expert geomorphic assessment.^25^^,^^28^ Oblique 3-D visualization in Google Earth Pro was used to enhance recognition of flow structures and frontal steepening, which are typical of active RGs. Secondly, kinematic consistency is required for validation. A candidate polygon is retained only if SBAS-InSAR deformation is spatially coherent and collocated with the mapped landform. Specifically, deformation must (i) occur within the polygon boundary, (ii) affect a substantial portion of the debris lobe, and (iii) persist over the multi-annual observation window. This requirement follows recommendations by Bertone et al.,^24^ who emphasize that isolated deformation patches or incoherent signals should not be interpreted as rock-glacier motion. In Figure 19A, coherent LOS displacement is confined to debris-mantled lobes, whereas adjacent bedrock slopes remain stable. The corresponding optical image (Figure 19B) confirms geomorphic alignment between deformation and rock-glacier morphology.

To further reduce operator bias, a stable-moving point comparison is implemented within each polygon. Internal points located within deforming lobes are compared against nearby stable reference terrain outside the active footprint. Accepted polygons must show near-zero long-term displacement at stable locations (within the ±5 mm background noise envelope) and consistent deformation at internal points. Similar internal consistency checks have been applied in recent SBAS-based periglacial deformation studies to distinguish true creep from atmospheric artifacts or transient slope processes.^28^^,^^38^ Beyond morphology and kinematics, we introduce quantitative topographic constraints to further enhance reproducibility. As shown in Table 5, confirmed rock-glacier polygons cluster within a narrow elevation range (∼4,896–5,097 m a.s.l.) and occupy moderate slopes (mean 18–24°). The regional mean elevation (5,092.76 m a.s.l.) and slope (18.45°) are consistent with permafrost-controlled creep environments reported in other Himalayan and alpine settings.^25^^,^^34^ Aspect analysis indicates dominant south-southwest to southwest orientations (mean 197–240°), suggesting preferential development under conditions favorable for debris-mantled ice preservation. This statistically constrained elevation-slope clustering demonstrates that accepted polygons are geomorphologically structured rather than randomly distributed, strengthening the rigor of the inventory.

**Table 5:** Topographic characteristics of confirmed rock glacier polygons in north Sikkim

Region	Mean elevation (m a.s.l.)	Mean slope (°)	Mean aspect (°)
North west Sikkim (original)	5,049.32	19.98	202.39
North east Sikkim (original)	4,896.36	23.71	240.12
North west (subset of north Sikkim)	5,096.68	18.72	197.29
North Sikkim (overall)	5,092.76	18.45	212.45

Areas showing deformation inconsistent with geomorphology are conservatively excluded from or re-delineated. Where kinematic and morphological evidence are incongruent, polygons are flagged as uncertain, following international best practices for rock-glacier inventories.^24^^,^^34^ Overall, this combined morphology-kinematics-topography framework ensures that rock-glacier polygons in Sikkim are accepted only when independent optical, InSAR, and terrain-based constraints converge, thereby minimizing subjectivity and enhancing reproducibility.

## Discussion

The abundance in activity of Sikkim’s RGs confirms RGs as a major cryosphere component in the Eastern Himalaya.^39^^,^^40^^,^^41^ First-order area-thickness scaling suggests a rock-glacier ice volume on the order of 2–3 km^3^ (with acknowledged uncertainty), implying substantial long-term water storage and a potential buffer of runoff under warming.^4^^,^^39^ By exploiting Sentinel-1 SBAS LOS time series with a conservative ±5 mm yr^−1^ activity threshold and optical refinement in Google Earth Pro, we produce a kinematics-informed inventory of 626 RGs exceeding the 504 features reported by Jones et al.^4^ demonstrating that InSAR reveals actively deforming ice-debris landforms often missed by optics alone in cloud-prone, rugged terrain.^24^ Independent comparison with the Jones et al. points shows partial spatial correspondence (≈72% near InSAR-based polygon and ≈28% far from InSAR-based polygons) shown in Figure 7, while a broader proximity analysis indicates that most legacy points cluster near our polygons, consistent with representation differences (points vs. polygons) and the inclusion of intact but inactive/relict forms in morphology-only inventories. Crucially, we quantified InSAR accuracy using paired time series from a nearby stable reference point and a rock-glacier point within the study area: after removing seasonal components and large-scale trends, the stable site retains a near-zero LOS trend with low residual variance, whereas the rock-glacier site preserves a persistent monotonic LOS deformation that exceeds the ±5 mm yr^−1^ threshold confirming that the detected signals are physical and that our SBAS processing and corrections (including regression-based removal of orbital ramps and height-correlated atmosphere) provide millimeter-level stability at reference locations while capturing meaningful creep at active sites where the limitations remain.

Temporal decorrelation during the monsoon and in vegetated areas reduces coherence; steep relief introduces layover/shadow and LOS-projection effects; and the 2022–2024 observation window may not capture interannual variability or decadal trends.^24^^,^^42^ We mitigated these through coherence-weighted SBAS processing, regression-based ramp/atmospheric correction, optical cross-checks, and explicit accuracy validation with stable vs. moving points; nevertheless, continued multi-geometry acquisitions and longer time series will further improve detection fidelity and trend characterization. Overall, our results align with regional work highlighting the hydrological importance of RGs and the value of standardized, activity-based inventories for the Himalaya. We provide the first comprehensive, InSAR-driven inventory for Sikkim, establish transparent overlap/proximity metrics relative to a legacy dataset, and show that integrating kinematics with optical interpretation substantially improves detection and delineation where optical methods alone are challenged. Routine Sentinel-1 monitoring will be valuable for tracking changes in RGs activity and for refining estimates of their contribution to mountain water resources.

### Limitations of the study

Although the multi-sensor approach improves the reliability of RG detection, some limitations remain. The analysis relies primarily on Sentinel-1 SAR data, whose spatial resolution may limit the detection of very small or slowly moving RGs. Additionally, the identification of RGs is mainly based on remote sensing observations and comparison with existing inventories, and extensive field validation could not be conducted due to the remoteness and difficult accessibility of the Sikkim Himalaya. Future studies integrating higher-resolution SAR datasets, optical imagery, and field-based observations could further improve the accuracy of RG mapping and deformation assessment.

## Resource availability

### Lead contact

Requests for further information and resources should be directed to and will be fulfilled by the lead contact, Ajay Kumar (ajay.kumar.c2021@iitbombay.org).

### Materials availability

All the materials of this study are available from the lead contact without restriction upon request.

### Data and code availability

• The datasets analyzed in this study are publicly available at https://doi.org/10.5281/zenodo.19419743.
• The original code supporting this study will be made available from the lead contact upon reasonable request.
• Any additional information required to reanalyze the data reported in this article is available from the lead contact upon reasonable request.

## Acknowledgments

The authors acknowledge the open access Sentinel-1 SAR data provided by the European Space Agency through ASF vertex, InSAR processing for this study was performed using PyGMTSAR, utilizing both raw and processed datasets available through the platform. The authors also acknowledge the rock glacier inventory shapefiles developed by Stephan Harrison and colleagues, which were used as reference data for comparison and validation in this study. In addition, glacier outlines from the Randolph Glacier Inventory were used to support the analysis. The authors further appreciate the open-access satellite and geospatial data initiatives that make such datasets freely available, enabling large-scale cryosphere and geomorphological research.

## Author contributions

A.K.: conceptualization, methodology, data processing, formal analysis, and writing – original draft preparation; A.M.: data analysis, writing – review and editing; G.S.: supervision, review, and editing of the manuscript.

## Declaration of interests

The authors declare no competing interests.

## Declaration of generative AI and AI-assisted technologies in the writing process

During the preparation of this work the authors used Chat GPT to enhance the linguistic ability of paper. After using this tool/service, the authors reviewed and edited the content as needed and take full responsibility for the content of the published article.

## STAR★Methods

### Key resources table

**Table:** 

REAGENT or RESOURCE	SOURCE	IDENTIFIER
Sentinel-1 SAR data (IW SLC)	European Space Agency Copernicus Program & Alaska Satellite Facility (ASF)	https://browser.dataspace.copernicus.eu/ https://search.asf.alaska.edu/
Copernicus DEM (GLO-30)	European Space Agency Copernicus Program	https://browser.dataspace.copernicus.eu/
Rock Glacier Inventory	Jones et al.^4^	https://doi.org/10.1016/J.SCITOTENV.2021.145368
Glacier outlines	Randolph Glacier Inventory (RGI)	https://www.glims.org/RGI/
Optical imagery	Google Earth Pro	https://earth.google.com

**Software & Algorithm**		

PyGMTSAR (version)	GitHub	https://github.com/AlexeyPechnikov/pygmtsar
QGIS (version)	Free and Open Source	https://qgis.org/
Python (version)	Python Software Foundation	https://www.python.org/

### Experimental model in life sciences and study participant details

Omitted as our study does not involve biological models.

### Method details

We employed an interferometric synthetic aperture radar (InSAR) time-series approach based on the Small Baseline Subset (SBAS) technique (A^16^ to detect and quantify slow-moving rock glaciers. The overall processing workflow is summarized in Figure 2, encompassing data pre-processing, deformation time-series analysis, and post-processing steps for identifying active rock glaciers.

#### InSAR data and processing

We used Sentinel-1 C-band SLC data from 2022 to 2024 and for processing employed GMTSAR to automate co-registration, interferogram formation, adaptive filtering, SNAPHU unwrapping, and SBAS time-series inversion.^43^ Topographic phase was removed using Copernicus (∼30 m) as shown in Figure 2. The methodology involved multiple sequential steps, described below, from SAR image preparation to the validation of results. The SBAS-InSAR processing was conducted using the PyGMTSAR framework with a short temporal baseline network (sbas_pairs(days = 12)) to minimize temporal decorrelation while maintaining network connectivity across the 2022–2024 observation period. Spatial phase unwrapping was performed using the SNAPHU algorithm within the GMTSAR workflow. To improve signal-to-noise ratio and suppress decorrelation effects in steep Himalayan terrain, a stability-guided landmask was constructed from the pixel stability function (PS Function). The PS Function was multi-looked using coarsen = (1, 4) and a wavelength parameter of 100 to enhance spatial aggregation and reduce speckle-related noise. Pixels exceeding a stability threshold of 0.3 were retained. The mask was intersected with valid DEM-derived topography (nearest-neighbour interpolation) and refined using morphological filtering (binary opening and closing with a 20 × 20 structuring element), followed by connected-component analysis to preserve only spatially coherent regions. This refined mask constrained the SBAS inversion to coherent and topographically valid pixels, thereby reducing phase-unwrapping artifacts and improving velocity reliability. Seasonal–Trend decomposition using Loess (STL) was subsequently applied to the SBAS time series assuming an annual seasonal cycle to suppress snow/monsoon-related periodic signals prior to trend estimation. Rock glacier activity was classified using a conservative threshold of ±5 mm yr^−1^, selected to exceed the effective background noise level of the masked SBAS velocity field and to ensure that only spatially coherent deformation patterns were interpreted as active creep. The uncertainty of the SBAS-derived velocity field was assessed using manually selected stable bedrock reference areas characterized by consistently high coherence and absence of geomorphic movement. The LOS displacement time series over these stable zones shows fluctuations within approximately ±5 mm without a systematic long-term trend, indicating that residual atmospheric effects, decorrelation noise, and unwrapping artifacts remain within this range after masking and STL-based seasonal correction. Therefore, the ±5 mm yr^−1^ threshold adopted to classify rock glacier activity corresponds to the effective background noise envelope of the processed dataset. Deformation exceeding this threshold exhibits spatial coherence and morphological consistency with rock glacier flow structures, providing confidence that classified “active” units represent genuine creep rather than measurement noise as shown in Figure 9. Time-series analysis yielded line-of-sight (LOS) velocity and cumulative displacement maps for the study period. Seasonality removal and trend correction. For each pixel, the LOS time series *y*_*t*_ was decomposed via seasonal-trend decomposition,

y_t_ = T_t_ + S_t_ + R_t_

and the seasonal component (ytno ^*Season*^ *= y*_*t*_ *- S*_*t*_*)*Residual orbital ramps and height-correlated atmosphere were then modeled and subtracted by coherence-weighted multivariate regression in map coordinates and topography. Let *φ(p*,*t)* be unwrapped phase at pixel *p*, epoch *t*; with *x*, *y* the projected coordinates and *H* the DEM height, we fit (independently per epoch)

φ(p,t) ≈ X(p) β_t,_ X(p) = [1, x, y, xy, H, Hx, Hy, Hxy, H^2^, H^2^x, H^2^y, H^2^xy]

using weights from coherence. The modeled nuisance $\phi_{\text{trend}}$ was removed to obtain the trend-corrected phase $\tilde{\phi }=\phi -{\hat{\phi }}_{trend}$. A stable reference pixel anchored each scene

Δφ(p,t) = φ̃(p,t) - φ̃(p_0_,t), d_LOS_(p,t) = λ4πΔφ(p,t),

ensuring milli-meter level stability at the reference and suppressing residual epoch-wise biases.

$$\mathrm{d}\text{Los}\left(P,t\right)=v_{\text{LOS}}\left(p\right)\left(t-t_{0}\right)+c+\varepsilon t$$

with optional temporal weighting by coherence. Pixels were classified as active where $\left|\nu_{Los}\right|\pm 5\text{mm}yr^{-1}$. Spatially coherent patches of active pixels were converted to polygons and refined in Google Earth Pro, requiring characteristic rock-glacier morphology (lobate/tongue shapes, transverse ridges/furrows, frontal steepening). This yielded an InSAR-detected, kinematics-informed polygon inventory. To verify the LOS deformation and remove residual ramps, we paired a stable reference point (88.9274°E, 27.3421°N) with a nearby subsiding rock-glacier point (88.9268°E, 27.3405°N). SBAS time series were decomposed with STL and the seasonal component was excluded; remaining long-wavelength artifacts (orbital/height-correlated atmosphere/incidence effects) were modeled and removed via a multivariate regression in x, y, and topography H (trend correction). After this correction and anchoring to the stable point, the stable series is flat with low residuals, while the rock-glacier series retains a persistent, centimetre-per-year–scale LOS trend, confirming that the detected signal reflects true creep rather than atmospheric or orbital noise. This stable–moving pair validation, together with Google Earth Pro geomorphic checks and comparison to,^4^ underpins the accuracy of our inventory and the scene-wide trend correction applied prior to mapping.

#### SAR data processing and SBAS time-series analysis

Surface deformation associated with potential rock glacier activity was investigated using Interferometric Synthetic Aperture Radar (InSAR) time-series analysis derived from Sentinel-1A C-band SAR imagery acquired in Interferometric Wide (IW) swath mode. Single Look Complex (SLC) scenes from both ascending and descending orbital tracks were utilized in order to improve spatial coverage and reduce geometric distortions such as layover and shadow that commonly affect radar observations in steep mountainous terrain.

Approximately 70 SAR acquisitions per orbit direction were obtained between April 2022 and September 2024, providing a nominal revisit interval of approximately 12 days. The datasets were processed using a Small Baseline Subset (SBAS) InSAR approach, which enables the extraction of long-term ground deformation signals while minimizing temporal and spatial decorrelation.

The processing workflow included the following steps.

1. Co-registration of SAR images to a common master scene.
2. Generation of interferometric pairs using small temporal and perpendicular baseline thresholds.
3. Removal of topographic phase using the Copernicus DEM (GLO-30).
4. Phase filtering and unwrapping.
5. Atmospheric noise mitigation and time-series inversion to derive Line-of-Sight (LOS) displacement rates.

This workflow allowed the generation of spatially continuous deformation maps and displacement time-series suitable for detecting slow surface creep associated with rock glacier movement.

#### Seasonal Trend decomposition (STL) of deformation signals

To improve detection of rock glacier creep signals, the InSAR time-series displacement data were further analyzed using Seasonal Trend decomposition using Loess (STL). This method separates the time-series signal into three components.

• Trend component, representing long-term deformation,
• Seasonal component, associated with periodic signals such as snow cover or seasonal moisture variations,
• Residual component, representing noise or unexplained variability.

By isolating the trend component, seasonal artifacts related to snowfall, snowmelt, and surface moisture were minimized. This step allowed more reliable identification of persistent deformation signals associated with rock glacier creep.

#### Rock glacier candidate detection

Potential rock glacier locations were identified by analysing spatial patterns of deformation derived from the SBAS time-series. A conservative deformation threshold of approximately ±5 mm yr^−1^ in the radar Line-of-Sight (LOS) was used to distinguish stable terrain from potentially active creeping landforms.

To avoid confusion with debris-covered glaciers, Randolph Glacier Inventory (RGI) outlines were used to mask glacierized regions prior to candidate selection. Areas showing consistent deformation signals outside glacier boundaries were considered potential rock glacier candidates.

#### Geomorphological validation using optical imagery

All candidate deformation areas were subsequently examined using high-resolution satellite imagery in Google Earth Pro to verify their geomorphological characteristics. The identification of rock glaciers followed commonly accepted geomorphological criteria, including.

• lobate or tongue-shaped landform geometry
• ridge-and-furrow surface morphology
• steep frontal slopes indicating accumulated debris
• evidence of downslope flow structures

Only landforms exhibiting both InSAR-derived deformation signals and characteristic rock glacier morphology were retained in the final inventory.

#### Inventory comparison and validation

The resulting rock glacier inventory was compared with the existing regional dataset reported by Jones et al.,^4^ which provides point locations representing mapped rock glacier fronts across the Himalaya. Spatial correspondence between the InSAR-derived polygons and the existing inventory points was evaluated to assess consistency between the two datasets. Additionally, deformation time-series were examined at representative locations to verify the presence of sustained displacement in active rock glaciers and near-zero displacement in stable reference areas.

### Quantification and statistical analysis

Quantitative assessment of deformation was performed using the SBAS-derived displacement time-series. Line of Sight displacement rates were calculated over the full observation period for each detected feature. The observed deformation rates ranged approximately from −500 mm yr^−1^ to +600 mm yr^−1^, reflecting the variability of surface motion across different rock glacier units. Stable reference locations showed minimal displacement, confirming the reliability of the InSAR measurements. Based on the combined InSAR and geomorphological analysis, a total of 626 active rock glaciers were identified across the Sikkim Himalaya, providing an updated assessment of rock glacier distribution and activity in the region.

To provide a systematic and reproducible noise characterization in response to this concern, four stable reference pixels (P1–P4) were selected on exposed bedrock surfaces based on objective, pre-defined criteria specifically, SBAS coherence γ ≥ 0.35 maintained throughout the full observation period and no detectable surface change in multi-temporal optical imagery ensuring that the reference sample reflects stable bedrock conditions rather than a subjective manual selection as shown in Figure 11. The standard deviation of LOS displacements was computed independently for each pixel and then pooled as a band-wise spatial mean (P5, *n* = 68 acquisition epochs), yielding a formal noise floor of σ_noise = 3.06 mm; the per-pixel values range narrowly from 3.14 mm (P2) to 3.62 mm (P3), confirming spatially consistent noise levels across the reference area (Table 3; Figure 11). The applied detection threshold of ±5 mm yr^−1^ corresponds to approximately 1.6σ above this noise floor which conservatively exceeding the background noise envelope while the rock-glacier point of interest exhibits a cumulative LOS displacement of ∼400 mm over 2.5 years (∼160 mm yr^−1^), yielding a signal-to-noise ratio of approximately 52, which unambiguously confirms that the observed displacement signal cannot be attributed to atmospheric artifacts, orbital ramps, or any other noise source characterized by the stable bedrock reference pixels.
